# Changes in the concentration of air pollutants before and after the COVID-19 blockade period and their correlation with vegetation coverage

**DOI:** 10.1007/s11356-020-12164-2

**Published:** 2021-01-14

**Authors:** Manguo Zhou, Yanguo Huang, Guilan Li

**Affiliations:** Jiangxi University of Science and Technology School of Electrical Engineering and Automation, Ganzhou, China

**Keywords:** COVID-19, Air quality, Vegetation cover, Lockdown, Correlation coefficient, Air pollution

## Abstract

In order to control the spread of COVID-19, China had implemented strict lockdown measures. The closure of cities had had a huge impact on human production and consumption activities, which had greatly reduced population mobility. This article used air pollutant data from 341 cities in mainland China and divided these cities into seven major regions based on geographic conditions and climatic environment. The impact of urban blockade on air quality during COVID-19 was studied from the perspectives of time, space, and season. In addition, this article used Normalized Difference Vegetation Index (NDVI) to systematically analyze the characteristics of air pollution in the country and used the Pearson correlation coefficient to explore the relationship between NDVI and the air pollutant concentrations during the COVID-19 period. Then, linear regression was used to find the quantitative relationship between NDVI and AQI, and the fitting effect of the model was found to be significant through *t* test. Finally, some countermeasures were proposed based on the analysis results, and suggestions were provided for improving air quality. This paper has drawn the following conclusions: (1) the concentration of pollutants varied greatly in different regions, and the causes of their pollution sources were also different. The region with the largest decline in AQI was the Northeast China (60.01%), while the AQI in the southwest China had the smallest change range, and its value had increased by 1.72%. In addition, after the implementation of the city blockade, the concentration of NO_2_ in different regions dropped the most, but the increase in O_3_ was more obvious. (2) Higher vegetation coverage would have a beneficial impact on the atmospheric environment. Areas with higher NDVI values have relatively low AQI. There is a negative correlation between NDVI and AQI, and an average increase of 0.1 in NDVI will reduce AQI by 3.75 (95% confidence interval). In the case of less human intervention, the higher the vegetation coverage, the lower the local pollutant concentration will be. Therefore, the degree of vegetation coverage would have a direct or indirect impact on air pollution.

## Introduction

At the beginning of 2020, the new coronavirus broke out and spread rapidly around the world, which had a major impact on global public health and the economy. In early January 2020, the new type of coronavirus called Severe Acute Respiratory Syndrome Coronavirus 2 (SARS-CoV-2) was confirmed as the pathogen of COVID-19 in China (Lu et al. 2020). Wuhan broke out and quickly swept the world. Currently, countries around the world are actively taking various measures to manage and prevent the further spread of COVID-19. Wuhan would implement lockdown measures within 3 weeks after the outbreak (Lin et al. 2020), and countries such as France and Switzerland closed their borders in mid-March (Kinross et al. 2020). After the Chinese government implemented the travel ban and imposed the city blockade, the mobility of people had dropped by 69.85%, and the concentration of air pollutants had been reduced to a certain extent, indicating that the drastic reduction of population movement was closely related to the reduction of air pollution, especially the reduction of AQI, PM_2.5_ and CO was caused to some extent by personal activities. (Bao et al. 2020). After the outbreak of COVID-19 in early 2020, studies have shown that PM_2.5_ and NO_2_ in northern China have decreased by about 35% and 60%, respectively, but the secondary pollutant ozone concentration has increased by 1.5–2 times. (Shi et al. 2020). In addition, a real-time prediction system for the cumulative number of COVID-19 infections and infection trends has emerged. The system integrated the epidemic prediction model with real global pandemic data and considered the impact of environmental factors (temperature and humidity) and control measures to establish a more accurate global prediction system for COVID-19; a more accurate global prediction system for COVID-19 was finally established (Huang et al. 2020).

Climate change has affected the biological, physical, and chemical processes of the terrestrial ecosystem. As an important part of the terrestrial ecosystem, vegetation is more sensitive to climate change. Vegetation changes caused by climate change have directly affected the material and energy balance of the regional land-atmosphere interaction process. A large number of harmful substances produced by human activities and industrial production are discharged into the environment, which has a huge impact on the types of local vegetation and the extent of vegetation coverage. The pollutant gas emitted by humans enters the human body through the respiratory tract, which may link the concentration of air pollution with the risk of COVID-19 (Zhu et al. 2020). Relevant studies have shown that inhalable particulate matter is related to respiratory diseases, and every increase of 10 μg/m^3^ of PM_2.5_ increases the risk by about 1.5% (Luong et al. 2019). So, what is the connection between air pollutants and vegetation cover? Therefore, with the aid of the national lockdown policy implemented during the period of COVID-19, it is more valuable to study the relationship between vegetation cover and air pollution during the lockdown period.

As an important ecological parameter reflecting the growth of vegetation, vegetation coverage combines natural environmental changes with human activities and plays a key role in indicating changes in the ecological environment, simulation of surface processes, and hydro-ecological models. Studying the temporal and spatial characteristics of the Normalized Difference Vegetation Index (NDVI) of ecosystems in different regions cannot only reveal the response of vegetation activities in various ecosystems to climate change, but also provide strategies for different ecological regions to respond to climate change and provide ecological civilization construction. NDVI is a means to monitor the growth of surface plants using remote sensing. Among a series of vegetation indexes, NDVI is simple to calculate, easy to obtain calculated parameters, and has a wide monitoring range and is related to biomass, vegetation coverage, leaf area index, etc. The indicators to quantify the growth status of plants have good correlation and are widely used. NDVI is used to monitor vegetation growth and the degree of vegetation coverage based on the spectral values of remote sensing red and near-infrared bands. Its data sources are extensive, and the global vegetation index change data is one of the most commonly used long-term vegetation dynamic monitoring data sets.

In summary, this article first studied the impact of urban blockade on air quality during COVID-19 from the perspectives of time, space, and seasonality and discussed the factors that affect the concentration of pollutants. Second, this article used the Normalized Difference Vegetation Index (NDVI) to analyze the characteristics of air pollution in mainland China and used the Pearson correlation coefficient to explore the relationship between NDVI and air pollutants during and after the city blockade. In addition, in order to quantify the relationship between NDVI and AQI, this paper used linear regression to obtain the quantitative relationship between NDVI and AQI and obtained the significance and feasibility of the model’s fitting effect through *t* test. Finally, some countermeasures were proposed based on the analysis results, and suggestions were provided for improving air quality.

## Method and material

### Data collection and processing

**Fig. 1:**
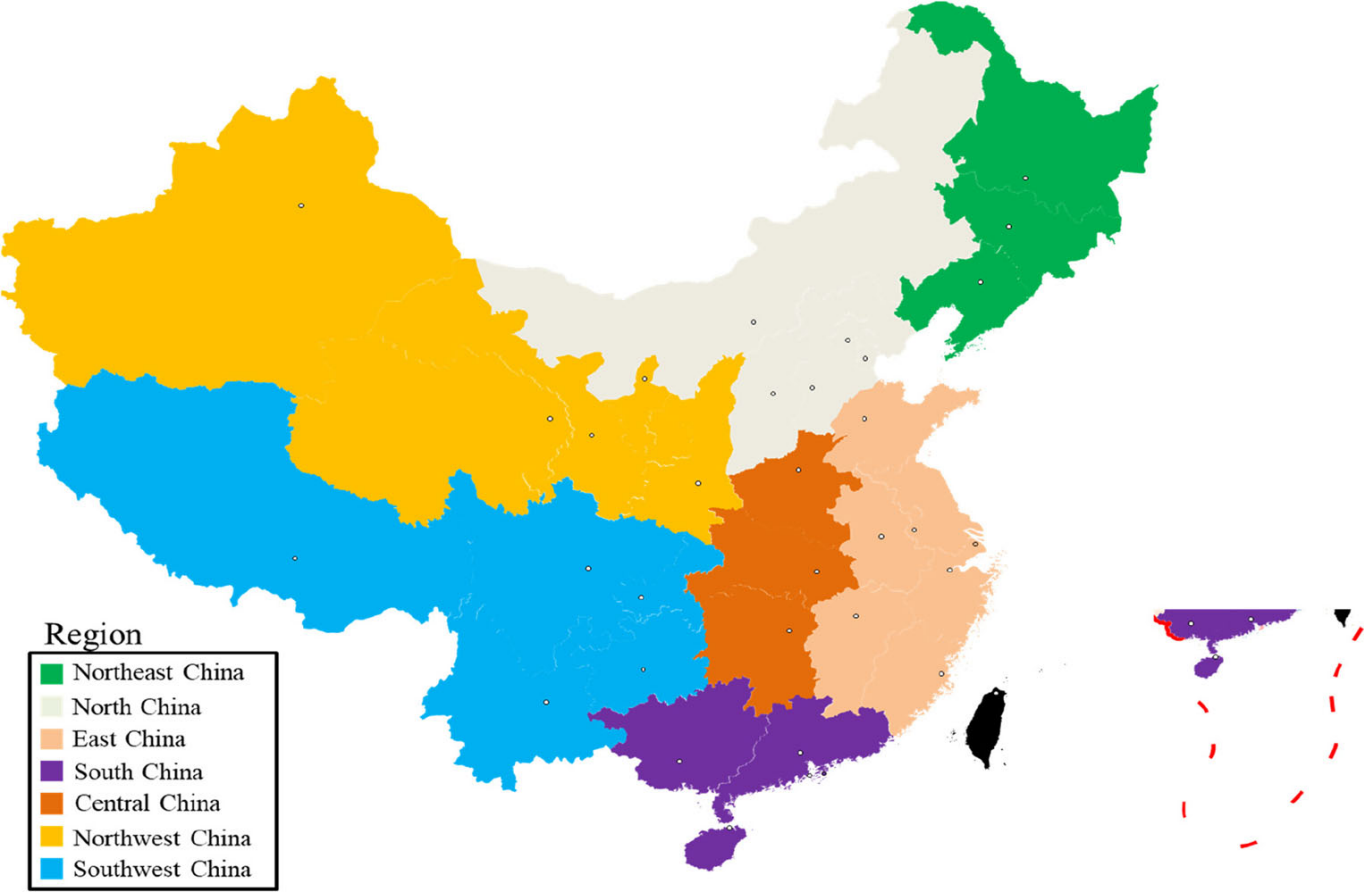
Seven major regions of mainland China based on factors such as geography and climate

The air quality data (including AQI, SO_2_, CO, PM_2.5_, PM_10_, O_3_, NO_2_) of 341 cities across the country studied in this paper came from the National Urban Air Quality Real-time Release Platform of China Environmental Monitoring Station (http://www.cnemc.cn/) and updated daily (MEE 2016); the research period was from January 2014 to June 2020. These cities covered most areas of mainland China, and the climate and vegetation characteristics of different regions varied greatly (Kuerban et al. 2019; Zhao et al. 2019). Considering the implementation of nationwide city lockdowns due to the rapid spread of COVID-19, this article focused on analyzing air pollution data from January 1, 2020 to June 31, 2020. In addition, we also selected the data for the same period from 2014 to 2019 for comparison. The spatial correlation of pollutants in the atmosphere is significant, that is, the concentrations of pollutants in similar regions are statistically closer (Wang et al. 2015). Therefore, this article divided the entire continent into seven regions, specifically based on geographic location and climatic conditions. According to the basic basis of seven physical geographical divisions, mainland China was divided into seven regions: North China, East China, South China, Central China, Northeast China, Southwest China, and Northwest China (Fig. 1). After removing the abnormal and missing values of the air quality index and pollutant concentration, the daily and monthly averages of different regions were calculated.

**Fig. 2:**
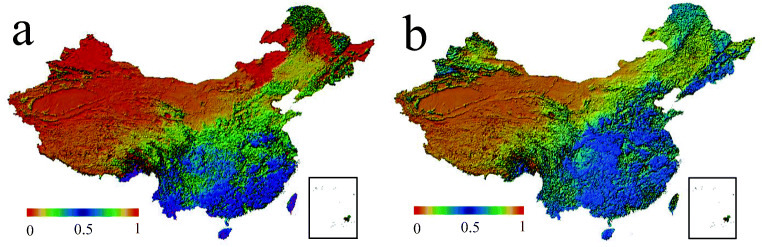
NDVI image stitched by MODIS, the average value of NDVI after removing negative values, the value range is 0 to 1. The higher the index, the richer the local vegetation coverage

This article obtained NDVI data through two channels, namely the Google Earth Engine (GEE) cloud platform and the NASA official remote sensing image data center. This article selected the MOD13A3 data set from NASA’s official remote sensing image data center, with a spatial resolution of 1000 m and a time period from February 2020 to May 2020. First of all, this article intercepted and covered all the images of mainland China and selected all the images during the vegetation growing season (February to June). Secondly, the median filtering method on the time series was used to fuse the monthly data sets into cloudless images with full coverage and balanced quality. Finally, image cropping and stitching were performed, and the synthesized images were checked one by one, and the synthesized images with low spatial coverage caused by the insufficient spatial coverage of the data set were eliminated. In addition, this article also used the Landsat 8 Collection 1 Tier 1 composite data provided by the GEE cloud platform for experiments. The NDVI is generated based on the near-infrared and red bands ((NIR − red)/(NIR + red)) of each scene, and the value range is − 1.0 to 1.0 (Fig. 2).

### Analytical method

The Air Quality Index (AQI) describes the degree of cleanliness or pollution of the air and its impact on health. It has no dimensions. The new air quality evaluation standards issued by the Chinese government in March 2012 included six pollutant monitoring items: SO_2_, NO_2_, PM_10_, PM_2.5_, CO, and O_3_. The data is updated every hour (MEE 2012).

Equation (1) is the air quality sub-index of pollutant item x:

1 $$ {\mathrm{IAQI}}_{\mathrm{x}}=\frac{{\mathrm{IAQI}}_{\mathrm{H}}-{\mathrm{IAQI}}_{\mathrm{L}}}{{\mathrm{BP}}_{\mathrm{H}}-{\mathrm{BP}}_{\mathrm{L}}}\left({C}_{\mathrm{x}}-{\mathrm{BP}}_{\mathrm{L}}\right)+{\mathrm{IAQI}}_{\mathrm{L}} $$

IAQI_x_ is the air quality sub-index of pollutant item x; *C*_x_ is the mass concentration value of pollutant item x; BP_H_ and BP_L_ are the pollutant concentration limits that are similar to *C*_x_ high value and low value; IAQI_H_ and IAQI_L_ can be found from BP_H_ and BP_L_, respectively. They are the corresponding air quality sub-indexes (Table 1).

**Table 1:** Air quality sub-index and corresponding pollutant item concentration limit

IAQI	Concentration limits of pollutants									
	SO_2_ (24-h average, μg/m^3^)	SO_2_ (1-h average, μg/m^3^)	NO_2_ (24-h average, μg/m^3^)	NO_2_ (1-h average, μg/m^3^)^a^	PM_10_ (24-h average, μg/m^3^)	CO (1-h average, mg/m^3^)	CO (1-h average, mg/m^3^)	O_3_ (24-h average, μg/m^3^)	O_3_ (8-h average, μg/m^3^)	PM_2.5_ (24-h average, μg/m^3^)
0	0	0	0	0	0	0	0	0	0	0
50	50	150	40	100	50	2	5	160	100	35
100	150	500	80	200	150	4	10	200	160	75
150	475	650	180	700	250	14	35	300	215	115
200	800	800	280	1200	350	24	60	400	265	150
300	1600	b	565	2340	420	36	90	800	800	250
400	2100	b	750	3090	500	48	120	100	c	350
500	2620	b	940	3840	600	60	150	1200	c	500

^a^The 1-h average concentration limits of SO_2_, NO_2_, and CO are only used for real-time reporting, and the 24-h average concentration limits of the corresponding pollutants must be used in the daily report

^b^If the 1-h average concentration of SO_2_ is higher than 800 μg/m^3^, the air quality sub-index calculation is no longer performed. The air quality sub-index of SO_2_ is reported as the 24-h average concentration

^c^If the 8-h average concentration of O_3_ is higher than 800 μg/m^3^, the air quality sub-index calculation is no longer performed, and the O_3_ air quality sub-index is reported as the sub-index calculated by the 1-h average concentration

NDVI is the ratio of the difference between the reflection value of the near-infrared band and the reflection value of the red light band in the remote sensing image, and it is also one of the important parameters reflecting the vegetation coverage. As shown in Eq. (2).

2 $$ \mathrm{NDVI}=\frac{\mathrm{NIR}-\mathrm{RED}}{\mathrm{NIR}+\mathrm{RED}} $$

In this paper, the independent sample *t* test method was used for the significance test, and the NDVI and air pollutant data during the blockade period were analyzed, and the differences were classified as not significant (*P* > 0.05), significant difference (0.01 < *P* < 0.05), and very significant difference (*P* ≤ 0.01).

The test statistics were calculated according to the following Eq. (3):

3 $$ t=\frac{{\overline{x}}_1-{\overline{x}}_2}{s_{\mathrm{p}}\sqrt{\frac{1}{n_1}+\frac{1}{n_2}}} $$

Among them:

4 $$ {s}_{\mathrm{p}}=\sqrt{\frac{\left({n}_1-1\right){s}_1^2+\left({n}_2-1\right){s}_2^2}{n_1+{n}_2-2}} $$

${\overline{x}}_1,{\overline{x}}_2$: respectively, represent the mean of two samples

*s*_1_, *s*_2_: respectively, represent the standard deviation of the two samples

*n*_1_, *n*_2_: respectively, indicate the number of two samples

*s*_*p*_: combined standard deviation

Correlation is a non-deterministic relationship, and the correlation coefficient is the amount of linear correlation between research variables. Due to the different research objects, this paper selected simple correlation coefficients, also called correlation coefficients or linear correlation coefficients, which were generally represented by the letter *r* to measure the linear relationship between two variables. See Eq. (5) and Eq. (6).

5 $$ r\left(X,Y\right)=\frac{\mathrm{Cov}\left(X,Y\right)}{\sqrt{\mathrm{Var}\left[X\right]\times \mathrm{Var}\left[Y\right]}} $$

6 $$ \mathrm{Cov}\left(X,Y\right)=\frac{1}{n-1}\left(\sum \limits_{\mathrm{i}=1}^{\mathrm{n}}\left({X}_{\mathrm{i}}-\overline{X}\right)\left({Y}_{\mathrm{i}}-\overline{Y}\right)\right) $$

Among them, Cov(*X*, *Y*) is the covariance of *X* and *Y*, Var[*X*] is the variance of *X*, and Var[*Y*] is the variance of *Y*.

## Results

### Seasonal changes of air pollutants

**Fig. 3:**
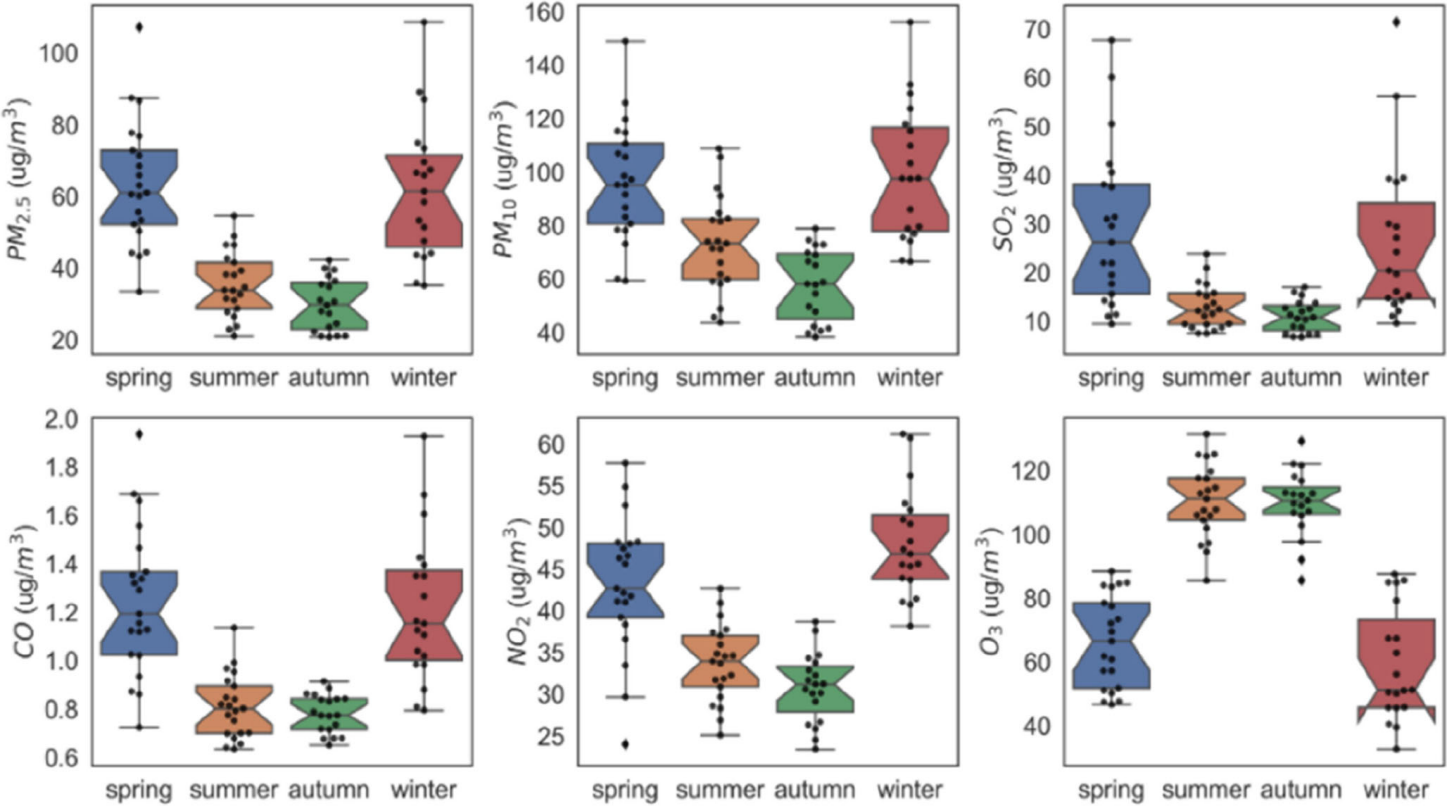
Seasonal characteristics of air pollutants from 2014 to 2019

Mainland China has a vast area, and the climate and temperature of different regions vary greatly. When the weather is colder, the gas diffusion rate is low. In winter, most areas are controlled by Mongolia-Siberian high pressure, with downdrafts dominated, and temperature inversion is prone to occur in winter, and pollutants are difficult to spread. In addition, there are relatively few plant leaves in cold weather areas, the ability to purify the air is relatively low, and the adsorption of atmospheric pollutants is relatively weak, so the air pollution index can reflect the local vegetation coverage to a certain extent. Therefore, it is necessary to study air pollutants and their concentration differences in different seasons. This article classified the air pollutants from 2014 to 2019 according to the season and analyzed the seasonality of several air pollutants (Fig. 3). Among them, the concentrations of PM_2.5_, PM_10_, SO_2_, CO, and NO_2_ were relatively high in winter and spring and relatively low in summer and autumn. The concentration of O_3_ was just the opposite of the change trend of the above-mentioned air pollutants. Its concentration was higher in summer and autumn and lower in spring and winter. Therefore, this article believed that there might be some seasonality in the concentration of air pollutants, which was also consistent with previous research conclusions (Escudero et al. 2019; Wang et al. 2020a; Lian et al. 2020).

It could be seen that the concentration of O_3_ in different regions was inversely proportional to the concentration of several other atmospheric pollutants (Figs. 3 and 5). Under the condition of sufficient solar radiation intensity, NO_2_ acts as the precursor of photochemical reaction and decomposes into NO and O(^3^P):

7 $$ {\mathrm{NO}}_2+\mathrm{h}\upnu \left(\lambda \le 430\mathrm{nm}\right)\longrightarrow \mathrm{NO}+\mathrm{O}\left({}^3\mathrm{P}\right) $$

8 $$ \mathrm{O}\left({}^3\mathrm{P}\right)+{\mathrm{O}}_2\longrightarrow {\mathrm{O}}_3 $$

9 $$ \mathrm{NO}+{\mathrm{O}}_3\longrightarrow {\mathrm{NO}}_2+{\mathrm{O}}_2 $$

It could be concluded from the Eqs. (7)– (9) that nitrogen oxides (NO and NO_2_) were an important prerequisite for the production of O_3_. In addition, NO could directly consume O_3_. The concentration of NO_2_ in the atmosphere was positively correlated with the concentration of NO, that was, lower NO_2_ concentration would lead to a decrease in NO concentration, thereby greatly reducing the possibility of NO and O_3_ reaction, causing O_3_ to accumulate, and ultimately leading to a continuous increase in O_3_ concentration. In the experiments conducted in this article, the concentration changes of nitrogen oxides (NO and NO_2_) and O_3_ had obvious negative correlation characteristics, especially in the cold winter. Photochemical reactions occurred in summer when solar radiation was strong, and this environment was more suitable for O_3_ accumulation. Therefore, the photochemical reaction during the daytime in winter was relatively weak, so a higher NO_2_ concentration in a specific range was more conducive to the consumption of O_3_, but a lower NO_2_ concentration would cause more O_3_ to be produced during the day, which could not be further effectively converted (Zhao et al. 2018a; Biswas et al. 2019). This was a good explanation for the significant increase in O_3_ concentration in different areas of mainland China after the Chinese government adopted epidemic prevention and lockdown measures (Fig. 5).

### Analysis on the temporal changes of air pollutants in different areas of mainland China before and after the blockade period

**Fig. 4:**
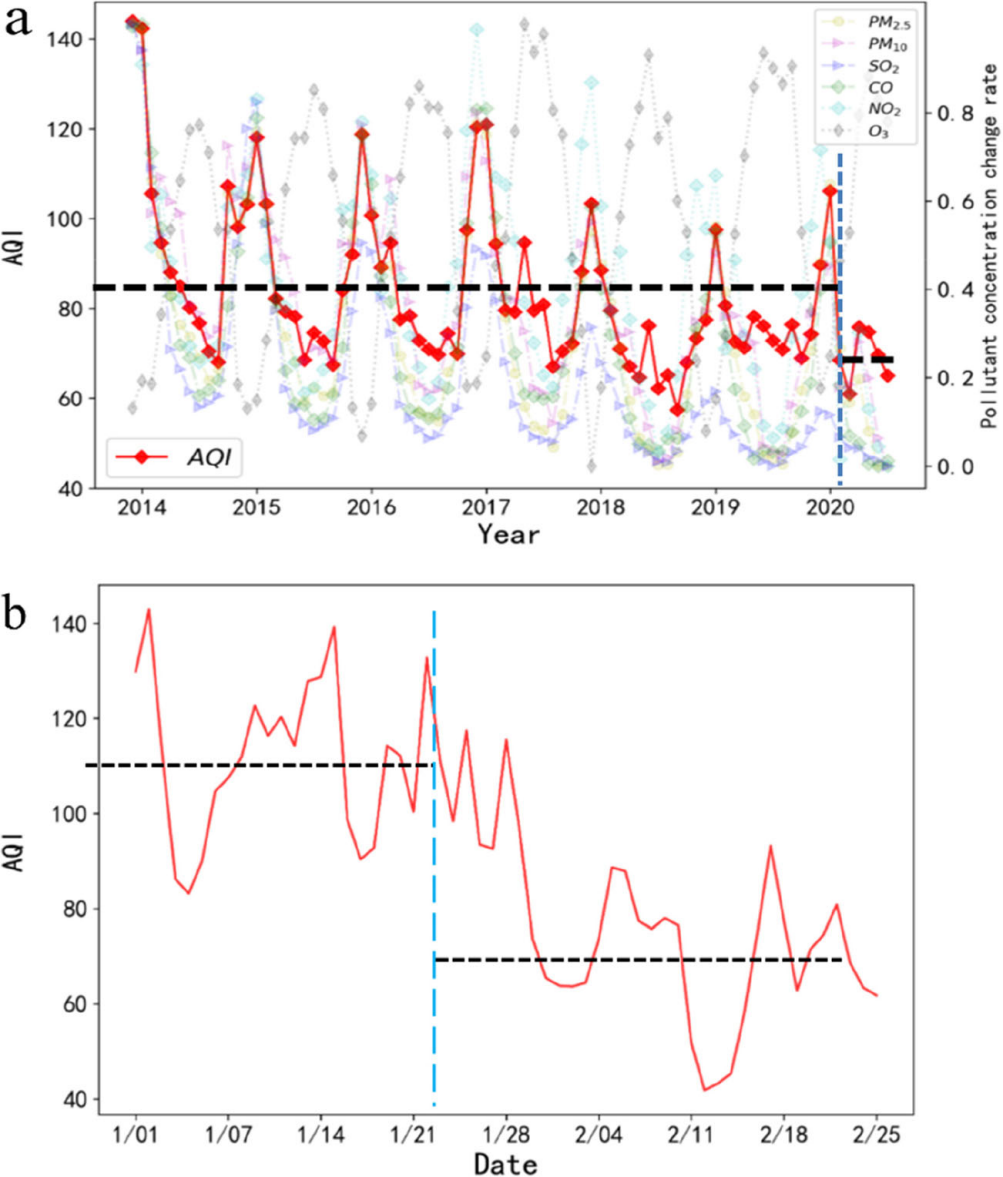
Temporal changes of air pollutants before and after the COVID-19 blockade. **a** The trend of the average air quality index in mainland China from 2014 to 2020. **b** The change trend of the average air quality index in Mainland China from January 1, 2020 to February 27, 2020

This article selected air pollutant data from 2014 to 2020, and the average AQI of various regions in China was generally on a downward trend. Although the overall air quality index had shown a downward trend in recent years, its downward trend had not been steady and still has a certain range (Fig. 4a). The lockdown period (the lockdown period is from January 23, 2020 to March 27, 2020) compared with the same period from 2014 to 2019, the AQI of different regions dropped by an average of 25.83%. Secondly, this article compared the lockdown period with the data before the lock-up period (selecting the date before the lockdown period is January 1, 2020 to January 23, 2020) (Fig. 4b), and it was found that the numerical change of AQI during the lockdown period was reduced by 39.03% on average compared with before the lockdown period. In addition, PM_2.5_, PM_10_, SO_2_, CO, and NO_2_ were positively correlated with changes in AQI and negatively correlated with changes in O_3_ (Fig. 2). Among these air pollutants, the concentration of PM_2.5_ dropped by 35.59%; the concentration of PM_10_ dropped by 38.52%; the concentration of SO_2_ dropped by 20.81%; the concentration of CO dropped by 31.10%; the concentration of NO_2_ dropped by 55.10%; and the concentration of O_3_ increased by 82.52%. It could be seen that the AQI in various regions of the country had dropped significantly; among the air pollutants, the concentration of NO_2_ had the largest decrease, and the concentration of O_3_ had the most significant increase.

According to research, due to the implementation of the city blockade policy resulting in suspension of work and production and traffic restrictions, the traffic volume in China had been reduced by about 70% (Xin Huang et al. 2020). The NO_2_ concentration obtained in this article showed the most obvious improvement during the city blockade of the COVID-19. The average concentration was reduced by about 55.10%. Therefore, NO_2_ might have a higher correlation with traffic pollution, which was more consistent with the previous research results (Lian et al. 2020; Wang et al. 2020a, b). PM_2.5_ and PM_10_ were the air pollutants with the largest decrease in concentration after NO_2_. PM_2.5_ mainly came from the fine particulate matter of combustion sources, including motor vehicle exhaust, flue gas emissions from power plants and chemical plants, and straw burning, etc. PM_10_ mainly came from motor vehicle dust, construction dust, other inorganic dust particles, etc. These two kinds of air pollutants also had a high relationship with industrial production and transportation. In addition, CO was mainly derived from incomplete combustion activities, such as fuel combustion and automobile exhaust emissions related to human activities. Therefore, the reasons for the decrease in the concentration of the three air pollutants of PM_2.5_, PM_10_, and CO could be considered to a certain extent as the result of the city blockade implemented by the government. However, the concentration of O_3_ had increased by 82.33%, and the increase was huge. According to “Seasonal changes of air pollutants”, the photochemical reaction in mainland China was relatively weak in winter, and measures such as shutdowns and traffic restrictions implemented by the government had effectively reduced nitrogen oxide emissions. Therefore, lower NO_2_ concentrations would greatly increase O_3_ concentrations. In addition, the reason for the increase in O_3_ concentration might be related to changes in the concentration of main air pollution and its meteorological conditions. Some articles (Zhang et al. 2016) had proved that O_3_ can exist for a longer time at a lower temperature, so that the concentration of O_3_ continued to accumulate.

### Analysis on the spatial difference of air pollutants in different areas of mainland China before and after the blockade period

**Fig. 5:**
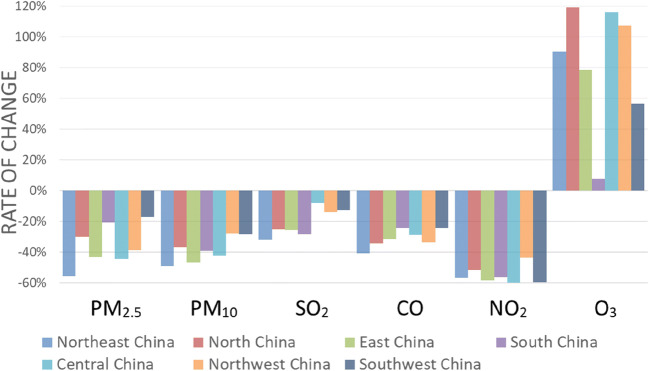
Changes in the concentration of different air pollutants before and after the lock-in period of COVID-19 in seven regions of mainland China

This article divided mainland China into seven main regions based on geographic environment and climate type. The air pollutant concentration changes in different regions before and after the lockdown period were quite different, and the main pollutants in different regions were also slightly different. The AQI in Northeast China decreased by 60.01%; the AQI in North China decreased by 30.48%; the AQI in East China decreased by 11.64%; the AQI in Central China decreased by 25.74%; the AQI in Northwest China decreased by 44.10%; and the AQI in Southwest China increased by 1.72%. It could be seen that the AQI in the Northeast China had changed most significantly, while the AQI in the Southwest China was almost unchanged. The study found that the higher the latitude, the greater the change in AQI, which was very similar to the vegetation coverage trend of mainland China. As the latitude decreases, the vegetation coverage became higher. In addition, the concentration of air pollutants in different regions also varied greatly before and after the blockade period (Fig. 5). The concentration of PM_10_ in various regions of the country had been significantly reduced, while PM_2.5_ was slightly different. The PM_2.5_ changes in Northeast China, North China, East China, Central China, and Northwest China were relatively large, with an average decrease of 42.25%, while the decrease in PM_2.5_ in Southwest China and South China was relatively low, with a decrease of 18.93%. This article mentioned in “Analysis on the temporal changes of air pollutants in different areas of mainland China before and after the blockade period” that PM_2.5_ and PM_10_ were related to the fine particulate matter of combustion source and dust and dust, respectively. Therefore, the reduction of industrial production and construction activities during the Lunar New Year holiday at the end of January 2020 would help reduce the concentration of PM_2.5_ and PM_10_, and the strict implementation of epidemic prevention measures could also greatly reduce emissions from transportation and industry. The PM_2.5_ values in Southwest China and South China were originally very small (33.100 μg/m^3^ and 22.167 μg/m^3^), indicating to a certain extent that the air quality in this area was better, and the reduction might be related to the vegetation coverage. In addition, the decline of CO and NO_2_ in different regions was basically the same, with an average decrease of 31.11% and 55.10%, respectively. During the blockade, vehicle exhaust and industrial production emissions were greatly reduced, which led to a reduction in NO_2_ and CO concentrations. At the same time, the change in SO_2_ concentration in the Southwest China and Northwest China was relatively small, with an average decrease of 13.35%. The populations of these two areas were relatively sparse, and the industrial capacity was relatively weak, so after the implementation of the city blockade, the concentration of SO_2_ in these two areas did not drop too much. Similarly, the concentration of O_3_ in Southwest China and South China had a small increase relative to other regions, with an average increase of 52.94%. It could be seen from “**Seasonal changes of air pollutants**” that the higher the temperature, the more intense the photochemical reaction of O_3_. The temperature in South China and Southwest China was higher than that in other regions. Although the content of nitrogen oxides was lower during the blockade, the conversion of O_3_ was weakened, but areas with relatively high temperatures react violently, which also reduced O_3_ to a certain extent of concentration.

### Correlation analysis of NDVI, AQI, and air pollutants during COVID-19

**Fig. 6:**
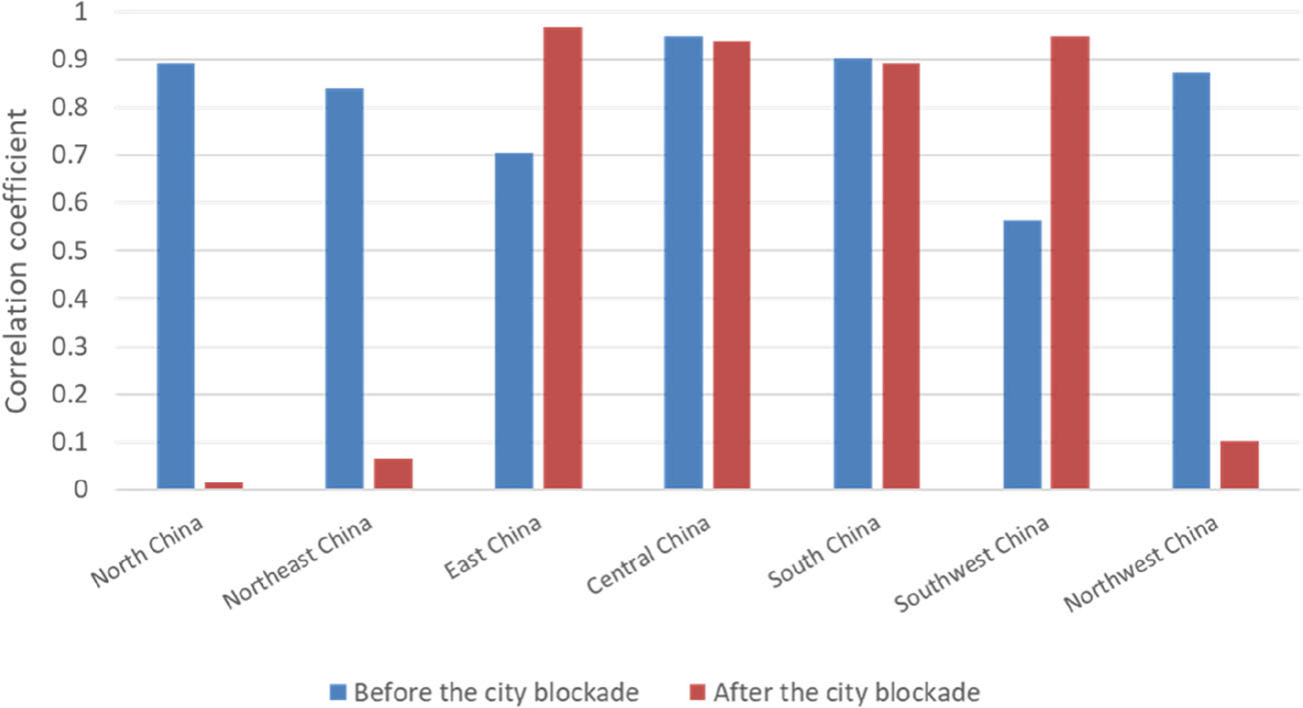
Correlation coefficients of NDVI and AQI in different areas of mainland China before and after the national lockdown

**Fig. 7:**
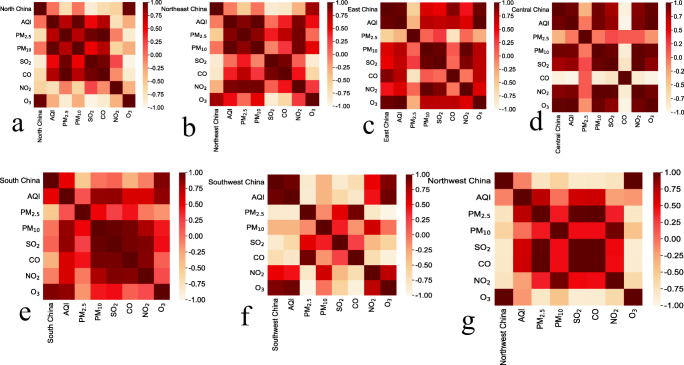
Correlation coefficients between NDVI and air pollutants in different regions of mainland China after the city was blocked. The correlation coefficient ranges from − 1 to 1. The closer the correlation coefficient is to 1, the stronger the positive correlation; the closer the correlation coefficient to − 1, the stronger the negative correlation; the closer the correlation coefficient is to 0, indicating that there is almost no correlation between the two. **a** North China; **b** Northeast China; **c** East China; **d** Central China; **e** South China; **f** Southwest China; **g** Northwest China

NDVI and AQI in different regions of mainland China before and after the lockdown period (Fig. 6), the correlation coefficients of NDVI and AQI in different regions of the country were quite different. After the implementation of the city blockade across the country, the correlation coefficients between NDVI and AQI in East China, Central China, South China, and Southwest China were still relatively high, while the correlation coefficients between North China, Northeast China, and Northwest China were relatively small. The correlation between air pollutants and NDVI values before and after the blockade in different regions of mainland China (Fig. 7) and the correlation coefficients of different pollutant concentrations were also quite different. First, after the implementation of urban blockades in different regions, the correlation coefficients of PM_2.5_, PM_10_, and NDVI had all been reduced to a certain extent. Among them, Central China had the largest decrease, with a decrease of 55.60%, while the correlation coefficient of PM_2.5_ in Southwest China did not change much, with a decrease of only 1%. The area with the highest correlation coefficient between PM_2.5_ and NDVI was Southwest China, while the area with the highest correlation coefficient between PM_10_ and NDVI was Central China. The reason for this result might be different vegetation types and main pollutants in different regions. SO_2_, CO, and NO_2_ also showed significant differences in different regions. For example, the correlation coefficients of SO_2_, NO_2_, and NDVI in South China were the smallest, being 0.05 and 0.08, respectively, which was almost irrelevant. This result might be caused by too little data to exclude the influence of factors such as errors. Or it was because the shutdown during the period of COVID-19 greatly reduced the emissions of these two pollutants, which made the data features unobvious. So, it was impossible to get a better correlation. In addition, the correlation coefficients between O_3_ and NDVI values in different regions of the country were relatively high. Generally speaking, the degree of air pollution would decrease with the increase of NDVI. Air pollutants such as SO_2_, CO, and PM_2.5_ were closely related to NDVI. Areas with high NDVI had relatively low concentrations of air pollutants. At the same time, areas with low vegetation coverage usually have more dust. Because vegetation has a certain blocking effect on dust, areas with low vegetation coverage are prone to produce particulate matter, such as PM_10_ and PM_2.5_. In addition, the increase in the concentration of pollutants such as SO_2_ and NO_2_ was also related to human activities.

The results in Table 2 correspond to Fig. 7, the confidence interval of the *t* test in this paper is 95%, As shown in the table, both NDVI and AQI are effective in different regions, so the experimental results obtained in this experiment can be considered credible. It could be seen that the correlation between AQI and NDVI in North China, Northeast China, and Northwest China was not obvious. From the results, it could almost be concluded that these three regions had almost no correlation with NDVI. And through actual analysis, AQI should indeed be related to NDVI, so there might be too little data to exclude the influence of factors such as errors. However, in addition to the above three regions, AQI and local NDVI in East China, Central China, South China, and Southwest China were highly correlated. According to the evaluation index, it could be seen that there was indeed a strong correlation between the AQI and the vegetation index in South China, Southwest China, East China, and Central China. However, there was basically no correlation between AQI and vegetation index in other regions, especially in North China and Northeast China.

**Table 2:** Significance test of AQI and NVDI in different regions of mainland China during the lockdown period was achieved by paired sample *t* test

Region	Standard error of the mean	Correlation	Sig. (bilateral)
North China	5.722	0.017	0.001
Northeast China	8.398	0.066	0.003
East China	6.630	0.968	0.002
Central China	5.613	0.938	0.001
South China	3.598	0.893	0.001
Southwest China	3.689	0.948	0.000
Northwest China	3.110	0.102	0.000

**Fig. 8:**
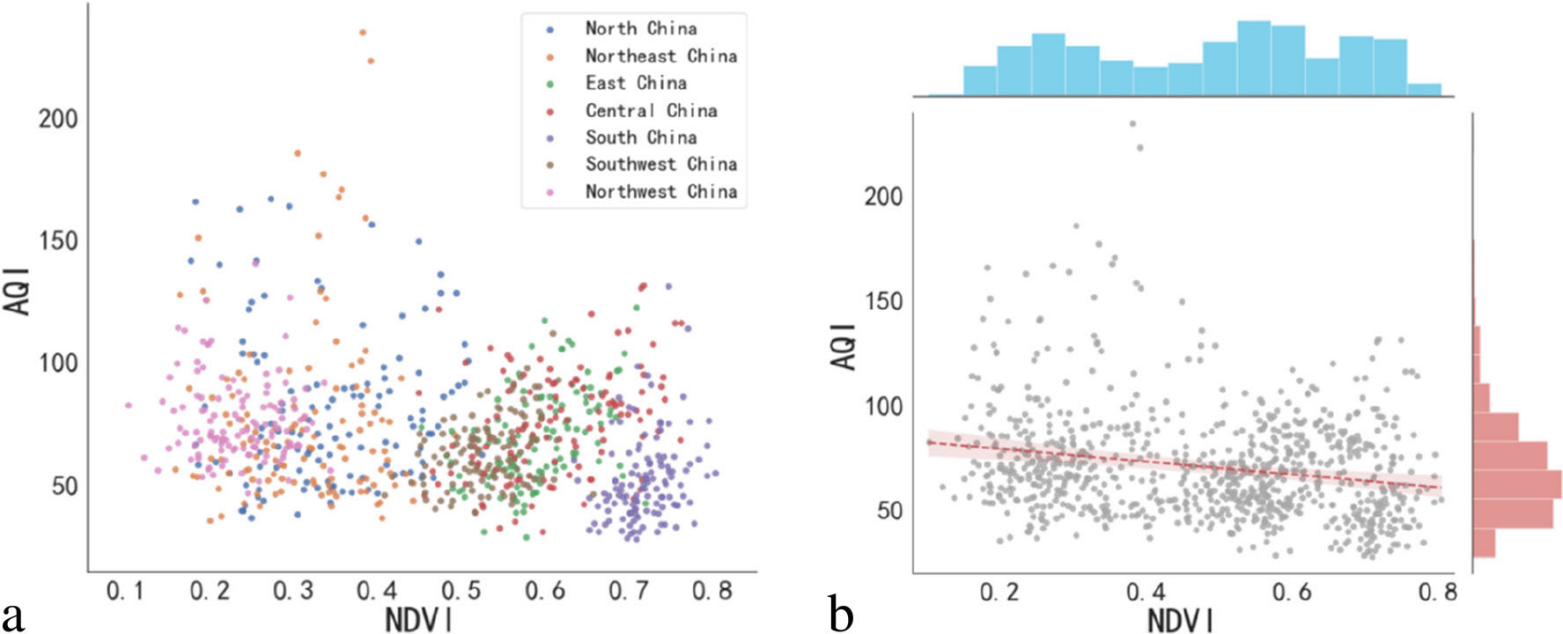
Correlation analysis between NDVI and AQI. **a** Data distribution of NDVI and AQI in different regions of mainland China. **b** NDVI and AQI have negative correlation characteristics

The result was that NDVI and AQI were negatively correlated, and an increase of 0.1 in NDVI would reduce AQI by 3.75 (95% confidence interval) (Fig. 8). Analyzing the fitting results, it could be seen that the fitting of AQI and NDVI used a straight line, combined with the actual environmental conditions, areas with rich vegetation coverage do had a greater degree of correlation with the environment. In the case of less human intervention, the higher the vegetation coverage, the lower the local pollutant concentration, which showed that the NDVI value and AQI were negatively correlated. This paper verified that the significance and correlation of NDVI and AQI were both valid and proved that the linear fitting made in this study was reasonable. However, during the period of the COVID-19, the implementation of the nationwide lockdown of cities had greatly reduced the concentration of many pollutants emitted by human. In the Northeast China, North China, and Northwest China, it was found that the fitting effect was not satisfactory, the distribution was very discrete, and the correlation was not high. This situation might be a problem with the data. Due to the small number of samples, it was impossible to distinguish the influence of noise, etc., so it could only be considered as the cause of the data, and the fitting was not valid.

## Discussion

This article first discussed the changes in air pollutant concentrations before and during the lock-in period of COVID-19 across China through the two dimensions of time and space, and then analyzed the relationship between NDVI and AQI in various cities during the lockdown period. As the Chinese government implemented a nationwide blockade, the concentration of air pollutants was less affected by humans, so it was meaningful to study the relationship between NDVI and AQI after the city is closed. After the implementation of the city blockade across the country, the changes in the concentration of pollutants in different regions had a large gap. The experimental results could explain to a certain extent that the main pollutants in different regions were different, and human consumption and production had a significant impact on the environment. In addition, this article also concluded that areas with higher vegetation coverage, that was, areas with higher NDVI values had relatively lower air pollutant concentrations. In the discussion in “Seasonal changes of air pollutants” and “Analysis on the temporal changes of air pollutants in different areas of mainland China before and after the blockade period”, the concentration of O_3_ was negatively correlated with the concentration of other pollutants (Fig. 3). This might be due to the photolysis of NO_2_ and the reduction of PM_2.5_ which accelerated the formation of O_3_ when solar radiation attenuated (Ma et al. 2019). However, in the actual atmosphere, there were many factors that change the O_3_ concentration. For example, volatile organic compounds (VOC) and CO can affect the change of O_3_ concentration. Among them, industrial production and transportation were the main factors for VOC generation (EEA 2019; Huang et al. 2011; Man et al. 2020). In addition, when the difference between the concentration ratio of VOC and NOx was too large, the steady-state cycle would have a large change (Chung et al. 1996). Finally, the data analyzed in this paper was in spring and summer when solar radiation was higher (intensity and daily duration), so it promoted the photolysis of NO_2_ (Escudero et al. 2019; Wang et al. 2020a, b).

**Fig. 9:**
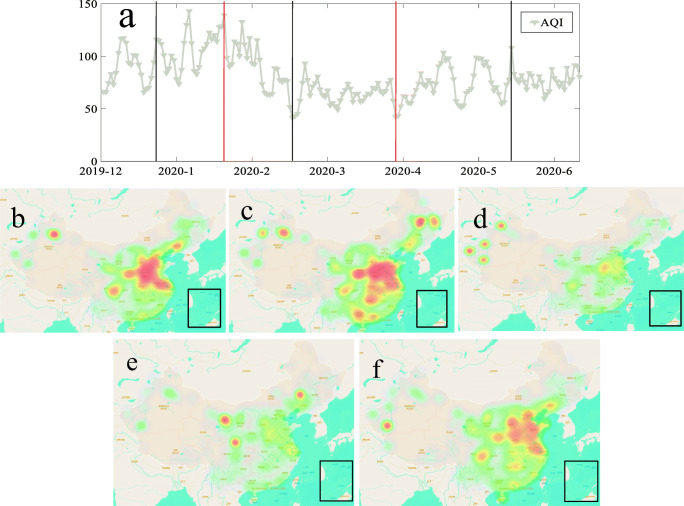
The trend of AQI changes in mainland China in two dimensions of time and space. **a** China’s average AQI change index from December 2019 to June 2020. There are five vertical lines in the figure, as shown in **b**–**f**. The two red vertical lines represent the start time and end time of the lockdown. **B** AQI distribution on December 24, 2019. **c** AQI distribution on January 23, 2020. **d** AQI distribution on February 17, 2020. **e** AQI distribution on March 27, 2020. **f** AQI distribution on May 13, 2020

China has a relatively complex topography, and the population of different regions is very different. The overall population density is large in the east and small in the west, large in the south and small in the north, and it is more concentrated in coastal areas. In order to intuitively understand the changes in AQI during the blockade of the COVID-19, this article drawn a heat map of AQI over time (Fig. 9). First, the area in the middle of the two red vertical lines represents the period of blockade of cities across the country. It can be seen that the AQI of the whole country has changed dramatically from the beginning of the blockade period to the end of the blockade period (Fig. 9c, d). The nationwide lockdown was implemented on January 23, and it can be seen that the AQI index in mid-February improved significantly. At the end of March, some cities gradually lifted their blockade, and the AQI of these cities gradually increased, such as Gansu, Shaanxi, Liaoning, and other provinces. With the gradual liberalization of cities, all walks of life are continuously put into production and operation. As of mid-May, it can be seen that the pollutant index in most parts of China is comparable with that before the blockade. Heavy industry and resources in Northeast, Central, and North China are relatively developed, such as steel in Hebei Province and coal in Shanxi Province. In addition, East China and South China are densely populated; traffic congestion in these areas, tires and brake wear, and tear will increase vehicle emissions (Han and Sun 2019) and cause air pollution. As a result, due to city closures and travel restrictions resulting in work stoppages and traffic restrictions, air quality has been significantly improved, and travel restrictions have greatly reduced air pollution in 341 cities in China.

However, this article found that different air pollutants vary greatly, and the rate of change of PM_10_ and NO_2_ is higher, which is largely due to the different sources of pollution. During transportation, vehicle exhaust and road dust would produce PM_10_ and NO_2_. The study also noted that low temperature had different effects on air pollution, which was significantly negatively correlated with SO_2_ and NO_2_, but positively correlated with PM_2.5_, PM_10_, and CO. The main source of SO_2_ was the combustion of sulfur-containing fuels (oil, coal, and diesel). Northeast China is China’s heavy industry base, while North China is an important resource extraction area. The light industry in East China and South China is relatively developed, so the blockade of these areas has made the improvement of sulfur dioxide more obvious. Central China was different from the Southwest China and Northwest China. The population density in the northwest was relatively low, while the southwest was mostly mountainous and forested. Therefore, the AQI index decline of these two regions was relatively low, but the northern regions (Northeast China, North China, Central China, East China) need to be heated in winter, and the burning fuel will produce some SO_2_. However, compared with 2014, the national average sulfur dioxide concentration during the blockade decreased by 35.33 μg/m^3^. This is not only due to China’s industrial upgrading, but also due to policy trends. In particular, the technological upgrading of the steel and power generation industries eliminated high-emission small and medium-sized coal-fired boilers. Rural heating has been transformed from coal and biomass to provide fuel for natural gas and electricity in recent years. Due to city closure measures and production shutdowns, the effect of NO_2_ control in different regions was very obvious (Wang and Su 2020). Changes in the ratio of NOx to VOC might also lead to an increase in O_3_ (Owoade et al. 2015), so the effectiveness of VOC control measures needs further research. Although NOx can cause the production of O_3_, the reduction of NOx is negatively correlated with the concentration of O_3_. Therefore, the quality and quantity conversion and connection between pollutants are very complicated. The production of secondary pollutants is affected by many factors. Its governance is not only related to emission reduction.

Respiratory diseases are usually more common in late winter and early spring (Lin et al. 2006; Cui et al. 2003), so the occurrence of COVID-19 may be partially affected by environmental and meteorological factors. For further research, we studied the relationship between NDVI and air pollutants during the period of COVID-19. A significant finding was that areas with lower NDVI values tended to have higher air pollutant concentrations. Although vegetation coverage was not suitable for the prevention and control of COVID-19, it helped prevent and control air pollution. Of course, China’s vegetation coverage cannot be judged by regions alone. The northern areas are often deciduous broad-leaved forests, while the southern areas are often evergreen broad-leaved forests. This leads to large changes in the NDVI value of northern China in winter and summer, which is greatly affected by the season. In addition, there was also a big gap in the vegetation coverage between urban and rural areas in the same area.

Analyze the problems of poor fitting results in North China, Northeast China, and Northwest China and analyze the causes of problems in these three areas. Northwest China is dominated by deserts and the annual NDVI value is low. The Northeast and North China are dominated by arable land. The vegetation coverage in this area has obvious periodicity. The NDVI value in summer and autumn is higher, and the higher vegetation coverage has a better effect on suppressing ground dust. The NDVI values in spring and winter in the region are low. Frequent farmland activities will produce a lot of dust, dust, and other pollutants exposed on the ground, which can easily cause dust to fly under the action of the wind and increase air pollution. Therefore, after analysis, it was found that the fitting effect between North China and Northeast China was not good. The reason might be that the forest branches and leaves were less in winter and spring, which could not stop the spread of pollutants in other regions. In addition, China would gradually resume industrial production and transportation at the end of March 2020, and the composition of air pollutants would also be affected by factors that were considered to change the original correlation between NDVI and AQI. Although several important confounding factors were adjusted, it was found that the fitting effect did not change much. Finally, the attempt to improve the fitting effect by improving the NDVI calculation method failed. The improvement effect was not obvious because the pixel resolution of MODIS was too low, and it was very easy to have mixed pixels. At this time, it could be considered that it was unreasonable for the pixels with negative NDVI to have no vegetation. The complex ground conditions also affected the calculated NDVI value of the pixel to a certain extent, so after removing the negative value, the average NDVI value was used to compare the simulation. The relationship between coalescence and meteorological factors had limited improvement in this study.

The analysis conducted in this study still has certain limitations. First of all, the vegetation coverage data selected in this paper were data from 341 cities in mainland China. The air pollution concentration and vegetation coverage data in different regions were selected as the average of several sampling points in the local cities. The different selection points might lead to a certain gap between the actual coverage and the experimental data. Even if the experimental results were valid for the purpose of proposing the hypothesis, it should not be concluded that the relationship between NDVI and AQI was a constant relationship. There would be a certain gap between the specific model parameters of NDVI and AQI in different regions. Second, the data obtained by MODIS cannot distinguish the types or types of vegetation. It is impossible to know from the MODIS data whether the type of green space was urban park, forest, agricultural land, or overgrown open space. Finally, due to lack of data, we cannot investigate the emergency measures of each city and all the factors and their long-term dynamic impact. It is not clear which parts of the emergency response in various regions of China were most effective in reducing air pollution. However, this article attempts to analyze air pollutants and vegetation coverage that may be important to protect medical professionals and control the spread of COVID-19. Despite these limitations, the results of this article have been verified to be reasonable.

The research in this paper found that there are differences in air pollution sources that affect air quality in different regions. The study found that air quality pollution may mainly be coal combustion, industrial exhaust gas, urban dust, automobile exhaust, and construction cement dust. The main air pollutants in different regions include PM_2.5_ and PM_10_, and they are closely related to AQI. Therefore, the prevention and control of fine particulate matter pollution are particularly important in controlling air pollution. In order to further improve the urban ambient air quality, the following suggestions are made:

1. In the research of this article, there are differences in air pollution sources that affect air quality in different regions, that is, air pollution influencing factors in different regions are different. In the control of air pollution, the treatment plans of different regions should be different and focused. It will be better to put forward improvement plans in a targeted manner.
2. Change the energy consumption structure and promote energy conservation and emission reduction; strengthen the supervision of industrial pollution source emissions, so that non-compliant emissions cannot be easily discharged; improve the reporting standards of engineering projects; strengthen the control of road construction and dust in urban construction; strengthen the management of road transportation vehicles; and strengthen road spraying and dust reduction.
3. Optimize industrial structure and layout. Relocate heavy industry industries, while vigorously developing financial services, medical services, education, and high-tech industries. To achieve the goal of not only reducing energy demand and pollutant emissions, but also ensuring sustained economic growth and full employment. And ease the contradiction between environmental protection and economic development.
4. Control the growth rate of automobiles, vigorously develop new energy public transportation, and promote green travel; further improve the subway lines, stagger trips, and reduce vehicle exhaust emissions.
5. Increase urban greening and beautification, increasing the area and coverage of green areas. Strictly control the sale and use of low-quality coal, especially coal used by urban residents for heating.
6. The regional transmission of pollutants is also a factor affecting urban air quality. Therefore, for the treatment of air pollution, attention should also be paid to the influence of external source transportation.

## Conclusion

Under the influence of national blockade measures, air quality across China has improved significantly. Compared with before the lockdown (106.16), the national average AQI (64.72) during the COVID-19 lockdown dropped by 39.03%; compared with the same period in 2014–2019 (87.26), the national average AQI during the COVID-19 lockdown (64.72) A decrease of 25.83%. In addition, the concentration of different pollutants (PM_2.5_, PM_10_, SO_2_, CO, and NO_2_) has also decreased to a certain extent, but the concentration of O_3_ has increased. Most of the reduction in the concentration of the first few pollutants is due to human factors, that is, the basic suspension of industrial production and transportation, thereby greatly reducing the emission of pollutants to the air. The increase in O_3_ concentration is a complex problem, which is affected by the photolysis of nitrogen oxides, as well as temperature and other environmental factors. Therefore, air pollution is a complex problem related to multiple factors. Although reducing the emission of one or several pollutants will help improve air quality, it may also bring new problems. In addition, the concentration of pollutants varies greatly in different regions, and the reasons for their composition are also slightly different. The Northeast China had the largest decrease in AQI (60.01%), while the region with the smallest change in AQI was in the Southwest China, which increased by 1.72%. The changes of NO_2_ in different regions are the same, while the changes of PM_2.5_, SO_2_, and O_3_ are quite different. Therefore, in the control of air pollution, the treatment plans of different regions should be different and focused, and it will be better to put forward improvement plans in a targeted manner. This paper also studied the correlation between NDVI value and pollutant concentration and quantitatively demonstrated the relationship between NDVI and AQI through linear regression. Higher vegetation coverage will have a beneficial impact on the atmospheric environment. Areas with higher NDVI values have relatively low AQI, and an average increase of 0.1 in NDVI will reduce AQI by 3.75 (95% confidence interval). In addition, the extent of vegetation coverage has a direct or indirect impact on air pollution and has certain guiding significance for the distribution and characteristics of pollution.

## Data Availability

Not applicable.
